# Conductive biological materials for in vitro models: properties and sustainability implications

**DOI:** 10.1007/s44164-025-00088-5

**Published:** 2025-04-24

**Authors:** Aleksandra Serafin, César R. Casanova, Arvind K. Singh Chandel, Rui L. Reis, Joaquim Miguel Oliveira, Maurice N. Collins

**Affiliations:** 1 Stokes Laboratories, Bernal Institute, School of Engineering, University of Limerick, Limerick, Ireland; 2 Health Research Institute, University of Limerick, Limerick, Ireland; 3 3B’s Research Group, I3Bs – Research Institute on Biomaterials, Biodegradables and Biomimetics, University of Minho, Headquarters of the European Institute of Excellence On Tissue Engineering and Regenerative Medicine, AvePark, Parque de Ciência e Tecnologia, Zona Industrial da Gandra, 4805-017 Barco Guimarães, Portugal; 4 ICVS/3B’s – PT Government Associate Laboratory, Guimarães, Portugal; 5 AMBER, University of Limerick, Limerick, Ireland

**Keywords:** Biodegradable materials, Conductive materials, In vitro models, Sustainability

## Abstract

The integration of conductive biological materials into in vitro models represents a transformative approach to advancing biomedical research while addressing critical sustainability challenges. Traditional materials used in tissue engineering and disease modeling are often environmentally detrimental, derived from non-renewable resources, and limited in their ability to replicate the dynamic properties of native tissues. Conductive biological materials bridge this gap by offering a unique combination of biodegradability, sustainability, and functional properties, such as bioelectricity and biocompatibility, that are essential for mimicking physiological environments. Herein, the development and current applications of biodegradable conductive materials, including advanced polymers such as polyaniline and polypyrrole, carbon-based nanocomposites, and renewable biopolymers derived from lignin and cellulose, are overviewed. These materials not only reduce the ecological footprint of biomedical research but also enable the precise simulation of electrical signaling in tissues, such as cardiac, neural, and muscular systems, thereby enhancing the physiological relevance of in vitro models. Their integration into three-dimensional (3D) tissue constructs, organ-on-chip platforms, and bioprinting technologies facilitates the development of patient-specific models, paving the way for personalized therapeutic and diagnostic applications. In addition to advancing biomedical precision, these materials align with global efforts to implement circular economy principles in research, promoting resource efficiency and waste reduction. By combining environmental responsibility with state-of-the-art functionality, conductive biological materials are redefining the future of in vitro 3D models and research, accelerating innovation in regenerative medicine, drug development, and disease modeling while fostering a sustainable framework for scientific discovery.

## Introduction

In recent years, sustainability has become a critical focus in biomedical research, with growing concerns over the environmental impact of traditional practices and materials [1]. The biomedical industry generates an estimated 5.9 million tons of waste annually, with a substantial portion being non-biodegradable or hazardous to the environment [2]. Materials such as petroleum-based polymers like polystyrene and polyethylene are one such example, as they pose significant environmental concerns due to their non-biodegradability and high carbon footprint [3]. These polymers dominate biomedical applications, particularly in cell culture platforms, scaffolds, and laboratory consumables, contributing substantially to plastic waste accumulation. It is estimated that more than 80% of biomedical plastics, such as those used in in vitro models, are derived from petroleum-based sources, contributing to environmental degradation [3]. For instance, polystyrene, which is commonly used in Petri dishes and cell culture plates, is a significant source of non-recyclable biomedical waste and contributes to microplastic contamination in marine and terrestrial [4], raising alarms about their long-term ecological consequences. With these statistics in mind, the push for more sustainable alternatives in research materials has never been more urgent.

For example, while polylactic acid (PLA), a biodegradable polymer derived from renewable sources such as corn starch, is often considered sustainable, its production requires intensive agricultural practices involving high water usage, fertilizer application, and land occupancy. Moreover, PLA biodegradation is not always straightforward, as it requires industrial composting conditions with specific temperature and humidity levels, which are not always available in standard waste management systems [2]. Therefore, a comprehensive life cycle analysis (LCA) approach must be applied to ensure that material choices contribute to sustainability rather than shifting the environmental burden to another life cycle stage.

Conductive biological materials present an innovative solution by providing environmentally friendly options that integrate biological systems with conductive properties, offering both sustainability and enhanced functionality for in vitro models [5]. Such type of materials holds the potential to reduce the ecological footprint of biomedical research while improving the accuracy and relevance of experimental models in disease modelling and therapeutic development.

Sustainable and conductive materials are of importance to the future of in vitro models due to their ability to address both environmental and scientific challenges associated with electrical stimulation of cells for enhanced tissue regeneration [6]. As the field progresses, there is a growing need for more accurate and physiologically relevant models to study complex biological systems which may often involve electrical stimuli for healing and regeneration or electrically induced triggering in the case of drug release systems [7]. Traditional in vitro models often rely on synthetic materials, which are now known to contribute significantly to environmental degradation. For instance, an estimated two million tons of medical plastic waste is generated annually, with much of it originating and attributed to biomedical research materials [8]. This growing environmental burden necessitates the development of sustainable alternatives.

Sustainable materials offer an eco-friendly solution, particularly biodegradable polymers and naturally sourced raw materials. These materials reduce the ecological footprint of biomedical research and can also be engineered to possess specific mechanical and biological properties, to display enhanced biocompatibility and tissue-like functionality [8]. A recent study demonstrated the use of plant-derived materials like cellulose nanocrystals for creating scaffolds that mimic the extracellular matrix, with potential as an in vitro model for tissue engineering and organ model trials [7]. By replacing petroleum-based plastics with sustainable materials such as these, a pathway to lower waste and reduced reliance on non-renewable resources can be achieved [9, 10]. However, using biologically derived materials alone may not be the solution as the whole life cycle of the materials must be assessed through robust and emerging life cycle analysis models which take into account the production, usage, and end of life of materials used in vitro models in a so-called cradle-to-grave approach. A significant challenge in applying LCA to biomedical materials is the high energy consumption required for processing and sterilization and the emissions associated with transportation, particularly for materials sourced from different geographical locations. Additionally, end-of-life disposal methods, such as incineration or landfill deposition, can negate the sustainability benefits if not managed appropriately [11].

Meanwhile, integrating conductive materials is essential for improving the functionality of in vitro models. As mentioned, conductivity is crucial for simulating electrical signaling in tissues, particularly for models of the heart, nervous system, and muscle tissues [12]. Research has shown that when incorporated into in vitro models, conductive polymers like polypyrrole and polyaniline can enhance cellular behavior, including differentiation and proliferation, by providing electrical cues that more closely replicate in vivo conditions [13]. These materials can also be used in organ-on-a-chip systems, where conductivity allows for better mimicry of the physiological environments necessary for drug testing, disease modeling, and sensing applications [14]. From a scientific perspective, developing sustainable and conductive materials offers significant advantages and associated challenges. These materials promote ecological responsibility and enable more accurate, reproducible, and versatile in vitro models that better mimic the dynamic electrical and mechanical environments found in living organisms. As the push for sustainability in research grows, these materials will be pivotal in shaping the future of biomedical science, offering a path toward more efficient, ethical, and environmentally conscious research practices [15].

The significant challenges that our modern society faces when treating different health problems are posed by the heterogeneity of the diseases and the need for cost-effective tools for pre-clinical and clinical research [16]. In pre-clinical research, the existing validation studies involve mostly 2D cell culturing methods, which are time-consuming and present only a narrow view of what is happening in the human body [17, 18]. Similarly, animal models are expensive and associated with poor reproducibility, thus not cost-effective and sustainable [19, 20]. In turn, in vitro 3D models are more cost-effective biomimetic microphysiological systems that have been developed as technological platforms to better recapitulate the physiological and pathological conditions of human diseases, thus potentially presenting a superior translational value [21, 22]. The in vitro 3D models involve multidisciplinary approaches combining materials science and engineering, biology, microfluidics, cell engineering and biology, and medicine. This new technological platform can help redesign research studies to be used for drug testing/discovery/validation and development of new diagnostic solutions as an example [23, 24]. As tunable biotechnological tools, in vitro 3D models can allow patient-specific models to be developed [25]. These can help predict individual responses to treatments and aid in the development of targeted therapies benefiting diagnostic and therapeutic interventions in personalized medicine approaches [25]. In order to better realize these technologies, (bio)materials currently used in the fabrication of in vitro models play a critical role as artificial extracellular matrices (aECM). The presence of such type of aECM components in in vitro models allows for cell adhesion, migration, and remodeling, enabling the in vitro formation of organized tissue structures and cellular phenotypes that more closely resemble those found in vivo [26]. In particular, hydrogel materials can better mimic the physiological complexity and microenvironment of native tissues, including biomechanical properties, ECM interactions, microarchitecture, signaling cues, conductivity, and reproducibility [27]. Thus, it is important to properly select the (bio)materials that best match the complexity of ECM of human tissues to be emulated. Of course, the (bio)materials selection process still poses major challenges not only due to the limited number of materials available and processibility, biodegradability, biocompatibility, and biofunctionality requirements but also due to other possible constraints associated with transferability to the clinics [28, 29].

Herein, we will overview the current developments and discuss the future perspectives involving conductive materials used as aECM and functionalization strategies in in vitro 3D models.

## In vitro 3D models

In vivo, cells exist in a dynamic 3D environment, interacting with extracellular matrix (ECM) components and neighboring cells. Traditional 2D cell cultures, while widely used, cannot fully capture these complex interactions, often leading to inaccurate or non-predictive data, necessitating further animal testing [30]. Alternatively, 3D in vitro models provide a more realistic approach to replicating tissue structure and function, making them essential for drug testing and biological studies. The significance of these models in these fields cannot be overstated. Hydrogels, which imitate the hydrated structure of the extracellular matrix (ECM), play a crucial role in these models [31]. Composite hydrogels, which combine synthetic and natural components, improve these systems’ mechanical strength and biological functionality. These advancements facilitate better cell interactions and create more physiologically relevant conditions. Despite considerable progress, challenges persist in fully replicating the complexity of human tissues. Recent developments in bioprinting and composite hydrogel technologies show promise but are still evolving [32–34].

**Fig. 1:**
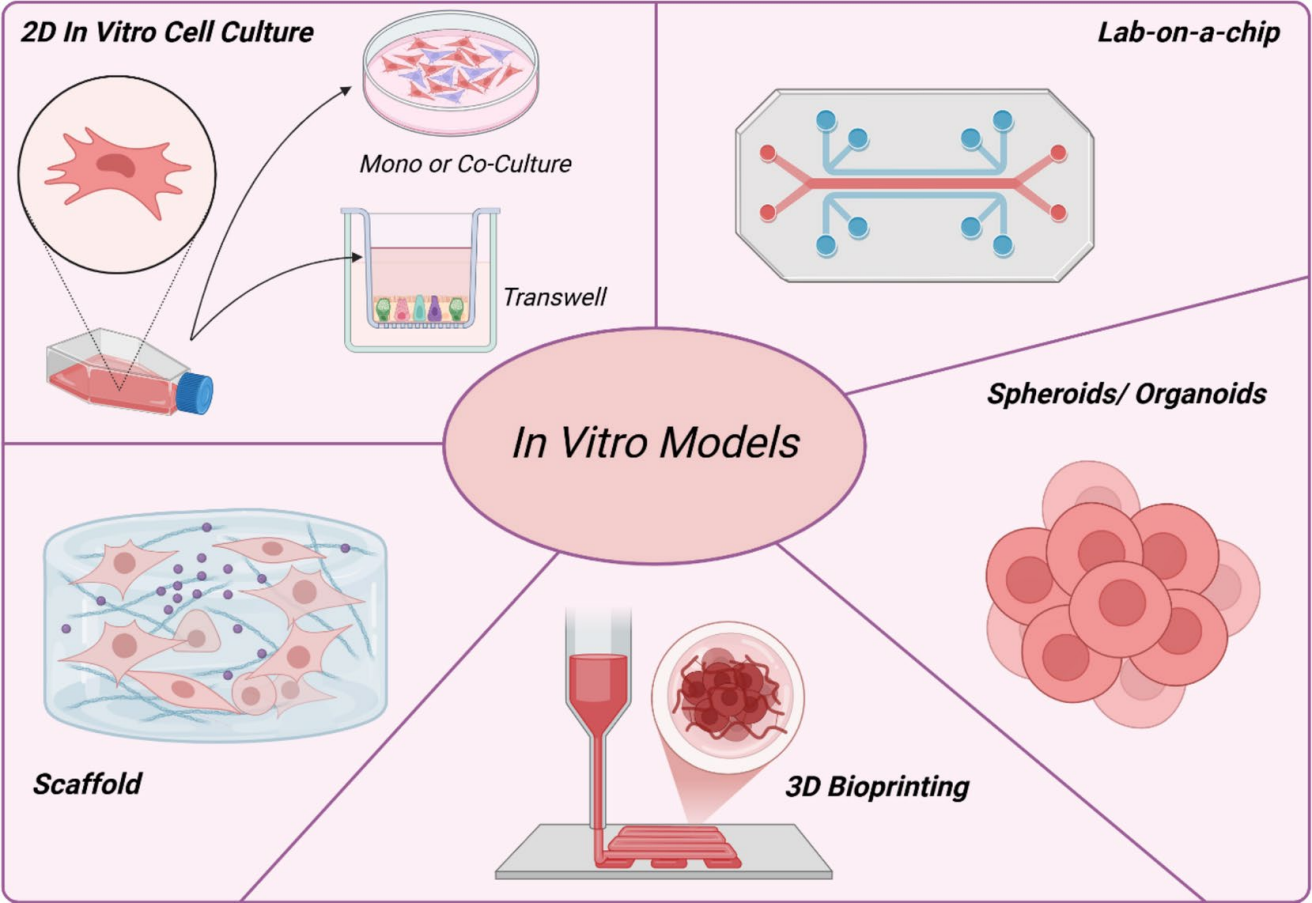
A schematic overview of the utilisation of in vitro models

Many in vitro 3D models have been created to replicate human tissues in a better biomimicry manner, see Fig. 1. These include cellular spheroids, organoids, cell-laden biomimetic constructs, and organ-on-chip technological platforms, amongst others. Among these, spheroids and organoids are especially popular because they enable cells to grow and self-organize in a three-dimensional environment, resembling actual tissues’ structural and functional characteristics [31]. Organoids, derived from stem cells or tissue fragments, can differentiate into various cell types. This capability enables them to serve as more accurate models for studying tissue behavior, drug screening, and disease [35]. Traditional extracellular matrix (ECM) -based matrices, like Matrigel®, have been used to support tissue structures but have limitations, such as variability between batches. Recent advancements have aimed at engineering biomaterials that offer better environmental control, allowing for more precise cell interactions and improved tissue formation [35, 36]. While organoids do not wholly replicate the complexity of natural tissues, their ease of assembly and ability to capture cellular diversity make them a valuable alternative to two-dimensional cultures and animal models [37, 38].

**Fig. 2:**
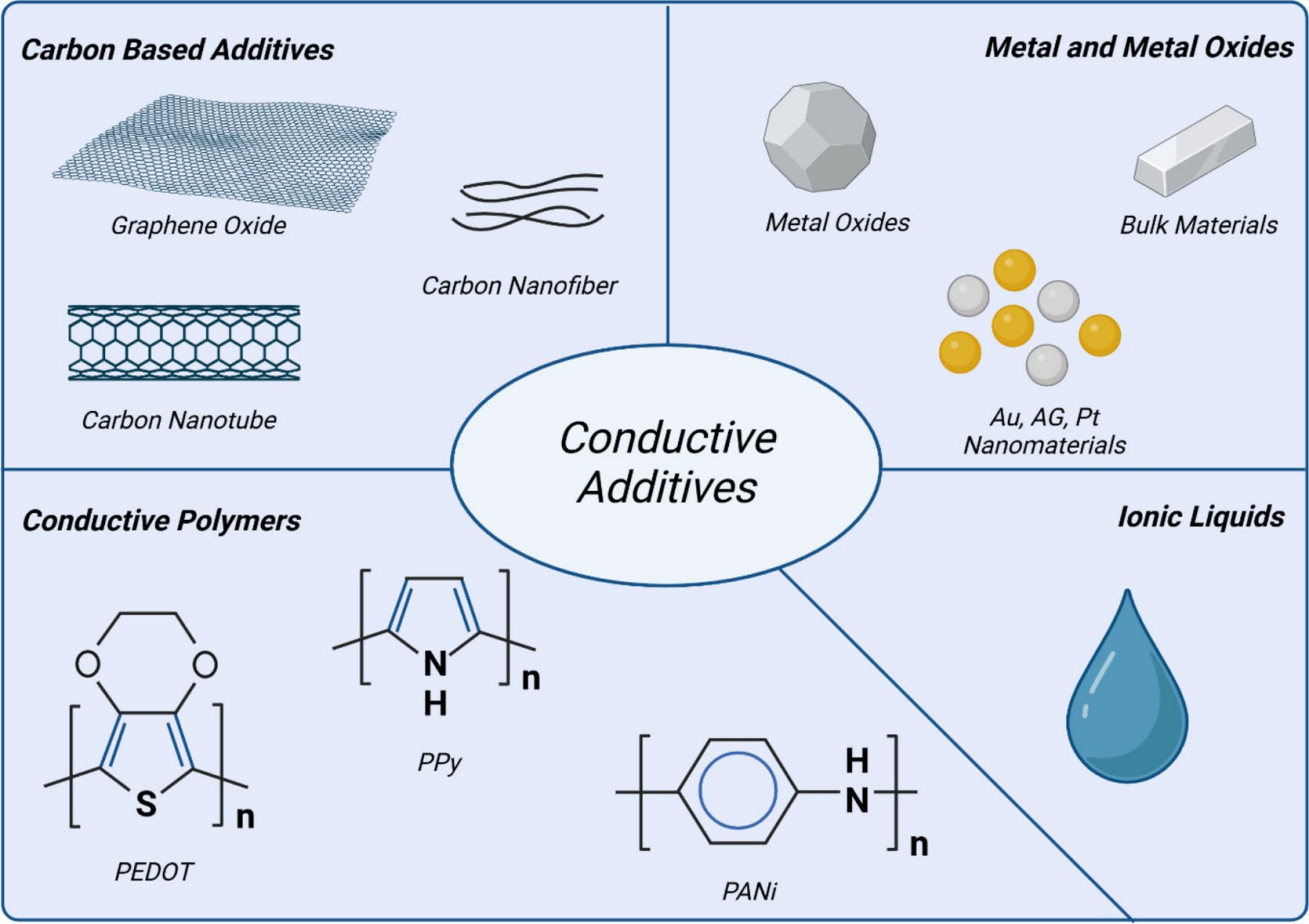
Schematic overview of conductive materials and additives

In organ-on-chip platforms, conductive materials are crucial in enabling real-time monitoring and electrical stimulation of tissue constructs as shown in Fig. 2. For instance, cardiac organ-on-chip models integrate microelectrodes fabricated using conductive polymers like polyaniline or polypyrrole to simulate the electrophysiological behavior of cardiac tissues. These materials help replicate native electrical signaling, enhance cell alignment, and support functional contractions of cardiomyocytes. A notable example includes a heart-on-chip system embedded with poly(3,4-ethylenedioxythiophene) polystyrene sulfonate (PEDOT:PSS) electrodes, facilitating continuous electrical stimulation and allowing for high-throughput drug screening application [39, 40]. Additionally, graphene-based biosensors have been incorporated into organ-on-chip devices to measure real-time electrochemical responses, improving the detection of cellular functions in neural and cardiac models [41]. These advances allow for better mimicking of physiological conditions and facilitate precise drug efficacy testing.

Conductive bioinks represent a significant advancement in bioprinting technologies, particularly for engineering neural and cardiac tissues. These bioinks, composed of biocompatible conductive polymers like polypyrrole or carbon nanotubes, enable printed constructs to support electrical conductivity while maintaining high cell viability. For example, a study demonstrated the use of a polypyrrole-alginate composite bioink to fabricate 3D-printed neural scaffolds with enhanced conductivity and improved neural differentiation [42–44]. In another application, graphene-infused bioinks have been utilized to print cardiac patches that mimic the conductive properties of native myocardial tissue, significantly improving electrical signal propagation in vitro [43]. Such conductive bio-inks are particularly beneficial for applications requiring electromechanical stimulation, such as muscle tissue regeneration and neural network formation.

**Fig. 3:**
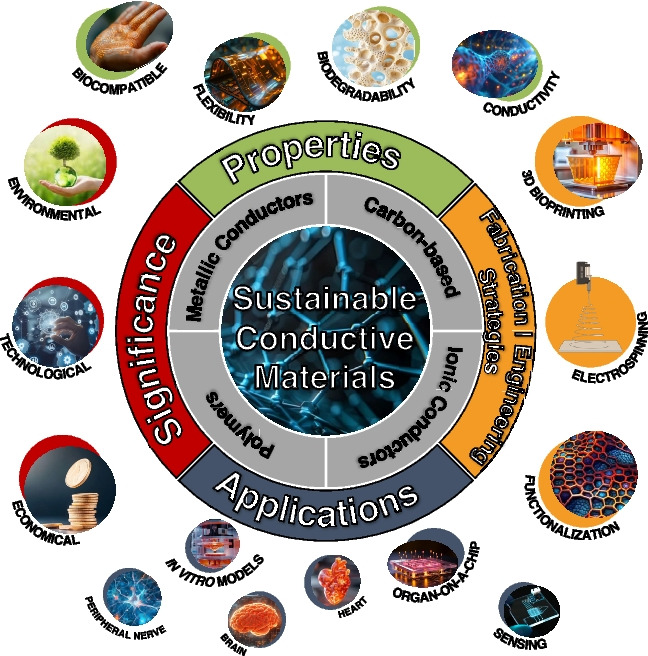
Integrative overview of sustainable conductive materials: Highlighting their significance, key properties, fabrication strategies, and applications in advanced tissue engineering and in vitro models. To enhance clarity and scientific visualization, selected graphical elements were sourced from BioRender and Adobe Stock

Composite hydrogels are increasingly recognized for their enhanced functionality and relevance in scientific matrices. Composite hydrogels provide a versatile and practical platform for advancing 3D in vitro models by incorporating elements such as drugs to influence cell behavior, additional ECM components to enhance mechanical properties, and biomolecules to improve biological activity [45]. The development of innovative sustainable materials for in vitro models has recently gained significant attention due to their potential to minimize environmental impact and enhance biocompatibility, see Fig. 3 [46]. These materials are derived from renewable sources and offer advantages such as biodegradability, reduced toxicity, and economic feasibility. Below are some key examples of sustainable materials being developed or used in in vitro models:

## Biodegradable polymers

Biodegradable polymers are increasingly used as scaffold materials in tissue engineering and in vitro models. Plant-derived materials are sustainable, biodegradable, and often biocompatible. They are gaining traction as alternatives to synthetic polymers in in vitro models.

Commonly utilized plant-based systems include the following:

### Polylactic acid (PLA)

Polylactic acid (PLA), derived from renewable resources like corn starch, is a biodegradable and biocompatible polymer, making it highly suitable for creating scaffolds for in vitro tissue models. PLA’s degradation products are non-toxic, ensuring they are safe for biological applications. Additionally, PLA can be tailored to achieve specific mechanical and chemical properties, allowing customized scaffolds that meet the unique requirements of different tissue engineering applications. This versatility and sustainability make PLA famous for producing scaffolds that support cell growth and tissue regeneration [46, 47].

### Cellulose and cellulose derivatives

Cellulose, obtained from plant fibers, is a widely utilized scaffold material due to its biocompatibility and biodegradability. Nanocellulose, a form of cellulose with nanoscale dimensions, has shown promise in creating highly porous and flexible scaffolds for 3D cell cultures, tissue engineering, and drug delivery [48].

### Agar and agarose

Agar and agarose, extracted from seaweed, are natural polysaccharides used to create hydrogels that mimic the extracellular matrix in vitro models. These hydrogels support cell culture by providing a customizable environment with adjustable stiffness and porosity. Agarose, known for its higher gel strength, is often preferred for tissue engineering and drug testing. The ability to tailor these materials makes them ideal for mimicking different tissue types and studying various biological processes. Agar and agarose are biocompatible, cost-effective, and widely used in biomedical research [49].

### Alginate

Alginate is a natural polysaccharide derived from brown seaweed or bacterial fermentation, consisting of β-D-mannuronic acid (M) and α-L-guluronic acid (G) units. It forms hydrogels through ionic crosslinking, particularly with divalent cations like calcium ions, making it ideal for tissue engineering and drug delivery applications. Biocompatible, biodegradable, and non-immunogenic, alginate supports cell encapsulation, providing a scaffold for cartilage, bone, and skin regeneration. It is also widely used in controlled drug release, particularly in wound care and cancer therapy, and for cell encapsulation in immune-isolation and cell therapy. Its moisture-retaining properties make it effective in wound dressings for exuding wounds [50].

Other potential sustainable systems include the following:

### Hyaluronic acid

Hyaluronic acid (HA) is a naturally occurring glycosaminoglycan found in the ECM of connective tissues, synovial fluid, and the eye’s vitreous humor. HA consists of repeating disaccharide units of N-acetylglucosamine and glucuronic acid and crucially may be derived from bacterial fermentation. Known for its high-water retention, biocompatibility, and viscoelasticity, HA is widely used in tissue engineering, particularly for cartilage repair, skin regeneration, and ocular applications. Its moisture-retaining properties are effective in wound healing and drug delivery, especially for anticancer drugs and growth factors. Additionally, HA is commonly used in cosmetic procedures, such as dermal fillers, to restore skin volume and reduce signs of aging [51].

### Collagen

Collagen is the most abundant protein in the human body, found in skin, bones, tendons, and cartilage. Typically extracted from animal sources such as bovine, porcine, and marine, collagen consists of a triple helix of polypeptide chains rich in glycine, proline, and hydroxyproline, which aggregate to form fibrils and fibers. Known for its biocompatibility, biodegradability, and mechanical strength, collagen is widely used in tissue engineering for skin, bone, and cartilage regeneration, supporting cell adhesion and differentiation. Collagen’s ability to naturally degrade through enzymatic action makes it ideal for biodegradable implants and scaffolds. It is also used in wound healing to promote tissue repair, in drug delivery systems for local applications like bone repair, and in cosmetic surgery for dermal fillers to correct wrinkles and scars [52].

### Gelatin

Gelatin is a natural biopolymer derived from collagen, primarily extracted from animal connective tissues such as skin, bones, and tendons. It is produced through the partial hydrolysis of collagen, resulting in long chains of amino acids, including a high glycine concentration, proline, and hydroxyproline. Gelatin is renowned for its biocompatibility, biodegradability, and non-toxic nature, making it highly suitable for various medical and pharmaceutical applications. One of its distinctive properties is its ability to form a gel when cooled and melt when heated, which proves invaluable in controlled drug delivery and tissue engineering. Also, gelatin’s high water absorption capacity makes it ideal for applications requiring hydration and moisture retention, such as wound healing and hydrating formulations [53].

## Environmental and economic benefits of using sustainable materials in in vitro models

Using sustainable materials in in vitro 3D models offers both environmental and economic benefits. Environmentally, sustainable materials, such as chitosan, alginate, and gelatin, are often derived from renewable sources and are biodegradable, reducing reliance on non-renewable resources and minimizing environmental waste. Their biodegradability ensures that waste generated during production or use breaks down naturally, reducing pollution and lowering the long-term impact on ecosystems. These materials are also more biocompatible and non-toxic, which prevents harmful chemical leaching and supports healthier biological processes in research. Additionally, producing sustainable materials typically involves fewer toxic emissions and lower energy consumption than synthetic alternatives, contributing to reduced environmental footprints [54].

Economically, sustainable materials can offer long-term, cost-effective ways to reduce waste management expenses and the need for expensive disposal processes. While initial production costs may be higher, the biodegradability of these materials leads to lower disposal costs and decreased reliance on costly petrochemical-based products. Moreover, the growing demand for eco-friendly products allows research institutions and companies to attract funding, grants, and public support. By investing in sustainable materials, businesses can drive innovation, open new markets, and strengthen their reputation in an increasingly environmentally conscious global market. These economic advantages and the potential for regulatory compliance and waste reduction make sustainable materials attractive for advancing in vitro research while supporting broader environmental and economic goals [55].

### Reduction in resource depletion

Sustainable materials, especially those derived from renewable sources, help reduce reliance on non-renewable resources. Traditional synthetic biomaterials often rely on finite petrochemical products, contributing to resource depletion. In contrast, materials like alginate, chitosan, and gelatin, which are derived from plants, algae, or animals, can be replenished naturally. This transition to renewable sources helps preserve fossil fuels and other finite natural resources, contributing to a more sustainable material supply chain [56].

### Biodegradability and reduced environmental pollution

One of the most significant environmental benefits of using sustainable materials is their biodegradability. Many sustainable biomaterials break down naturally through enzymatic or microbial action, leaving minimal environmental impact. This contrasts with synthetic polymers, which often persist in the environment for extended periods and require complex disposal processes. Materials such as chitosan and alginate degrade without producing harmful residues, significantly reducing the environmental footprint of biomedical research [57].

### Lower toxicity and chemical leaching

Sustainable materials are typically biocompatible and non-toxic, making them less likely to release harmful chemicals into the environment or the human body. This is particularly important in in vitro models, where the materials used for cell culture and tissue engineering should not interfere with biological processes. By using non-toxic, eco-friendly materials, researchers can reduce the risk of contamination, chemical leaching, or harmful byproducts, thereby ensuring safer and more sustainable scientific practices [58, 59].

### Support for circular economy

Sustainable materials help foster a circular economy, where resources are reused, recycled, and regenerated rather than discarded after use. For example, biodegradable biomaterials like chitosan can be used in in vitro models and then decomposed without leaving a long-lasting environmental impact. This supports waste reduction strategies and promotes the idea of “closing the loop” in materials usage, where products or components are continuously repurposed or safely returned to nature [60].

### Economic benefits of using sustainable materials in in vitro models

#### Long-term cost savings

While the initial cost of sustainable materials may sometimes be higher, they often lead to cost savings in the long run. The biodegradability of materials like alginate and chitosan reduces waste management and disposal costs, as they do not require complex or expensive disposal methods. Using renewable and biodegradable materials can also mitigate the rising costs of petrochemical-based products, which are subject to price fluctuations based on global supply and demand [61].

#### Market demand and competitive advantage

As consumers, regulators, and funding bodies increasingly prioritize sustainability, companies that incorporate sustainable materials in their in vitro models gain a competitive edge. By adopting eco-friendly practices, institutions can enhance their reputation and attract funding from government grants, research organizations, or private investors keen to support green initiatives. Furthermore, as demand for environmentally responsible products grows, businesses prioritizing sustainability are better positioned to meet this demand, opening new markets and opportunities for innovation [62].

#### Compliance with regulations

Governments and international bodies are increasingly introducing regulations to reduce environmental impact and promote sustainable practices. Using sustainable materials in in vitro models can help companies and research institutions comply with ecological standards, avoiding potential fines and penalties associated with non-compliance. These regulations are expected to become increasingly strict over time, and adopting sustainable materials now can position organizations ahead of possible future requirements.

#### Incentives and subsidies

Many governments and regulatory bodies offer financial incentives, tax breaks, or subsidies to businesses that invest in sustainable practices. By using renewable, biodegradable materials, companies involved in biomedical research can tap into these financial benefits, reducing overall operational costs. These incentives are designed to encourage the transition to sustainable practices and can provide a significant economic advantage in the long run [63].

#### Fostering innovation and new revenue streams

The growing focus on sustainability can spur innovation within the research and development sector. By exploring and developing new sustainable materials, companies can create novel products that cater to the increasing demand for environmentally friendly solutions in biotechnology and biomedical research. This opens up new revenue streams in industries such as green biotechnology, eco-friendly medical devices, and sustainable pharmaceuticals [64].

#### Reduced waste disposal costs

Traditional biomedical research materials often require expensive specialized disposal techniques, such as incineration or hazardous waste management. Sustainable materials, particularly biodegradable ones, reduce the need for such complex and costly waste management systems. This results in direct financial savings, particularly for large-scale research facilities and industries that generate significant waste during operations [36].

Incorporating innovative and sustainable materials into in vitro models offers several environmental and economic benefits, including waste reduction, cost savings, and enhanced biocompatibility. As materials science evolves, these sustainable alternatives will transform biomedical research’s future while supporting broader sustainability and eco-friendly initiatives [65].

## Conductive materials

Amongst the developed in vitro models, conductive in vitro platforms are beginning to emerge, particularly used for the examination of biological responses of naturally conductive tissues such as nerves or the heart. This pertains especially to the natural phenomena of bioelectricity emerging from the flow of ions within the examined cells and tissues, which play a critical role in cellular functions and activities. The application of these conductive materials in research can have many roles, ranging from providing a biomimicry environment of the native studied tissue, relaying the information from biological systems to the engineered system, as well as providing electrical impulses and stimuli to the studied cells in order to prompt desired cellular responses [66]. The altered conductivity of tissues also plays an important role in the pathological states of many tissues, including cancers [67], which can be levered to study disease models with the aid of the in vitro conductivity models.

The conductivity of such materials can be altered in many ways to achieve this. Generally, conductivity can be imposed onto sustainable biomaterials by means of conductive additives or conductive polymers. The former most often includes the addition of carbon-based materials such as carbon nanotubes (CNTs), graphene, carbon nanofibers (CNFs), and reduced graphene oxide (rGO). These materials exhibit high electrical conductivity, mechanical strength, and surface functionalization potential, making them ideal for biosensors, tissue scaffolds, and organ-on-chip models [68]. In neural tissue engineering, carbonized cellulose nanofibers have been found to support neurite outgrowth and improve electrical communication between neurons [69]. The amounts of these additives included in the biomaterial scaffolds have a great effect on the scaffold’s conductive capabilities. The greater the amount of additive suspended within the biomaterial scaffold, the greater the conductive potential, though at a certain point this behavior is tapered off when the precipitation threshold is achieved. Concerns regarding biodegradability and long-term biocompatibility have led to research into sustainable carbon materials derived from lignin and cellulose.

The conductive polymers can be added to the biomaterial scaffold in a similar fashion, with PEDOT:PSS, polyaniline and polypyrrole often used for this purpose, due to their unique combination of conductivity, biocompatibility, and mechanical flexibility. These polymers can be integrated into hydrogels, electrospun scaffolds, or films to enhance the bioelectrical properties of in vitro models. For example, PEDOT:PSS has been used in cardiac patches and neuronal scaffolds to provide electrical cues that mimic in vivo conditions [70]. Though as often as these additives have been utilized in tissue engineering endeavors, their use is now beginning to be questioned due to possible implications of the biodegradability of these materials. As such, wanting to still utilize the beneficial conductive capabilities of these materials, researchers have now begun to tailor these materials to the desired traits. One clear example of this is the elimination of the use of the PSS component in the PEDOT:PSS polymer. In its readily available form, PEDOT:PSS relies on the PSS component to enable its solubility in water, though this component is responsible for the poor biofunctionality and biodegradability of the material [39, 71]. Novel efforts have been made to eliminate this reliance on the PSS component, by synthetizing PEDOT nanoparticles (NPs). These NPs are synthetized via a mini-emulsion polymerization reaction by means of a surfactant with a negatively charged polyelectrolyte, with poly(diallyldimethylammonium chloride) solution (PDADMAC) and hyaluronic acid (HA) as two named examples [39, 72, 73].

Other nanoparticle additives include metallic nanoparticles, such as gold (Au), silver (Ag), and platinum (Pt) nanoparticles, which offer excellent electrical conductivity and have been widely used in biosensing, electrostimulation, and regenerative medicine. Gold nanoparticles (AuNPs), for instance, enhance electrical stimulation in cardiac and neuronal tissues, improving cellular adhesion, differentiation, and signaling [74]. Silver nanoparticles (AgNPs) have additional antimicrobial properties, making them suitable for wound healing applications in conductive hydrogels [75]. However, toxicity concerns require careful control of particle size, concentration, and surface modifications.

Ionic liquids, particularly bio-ionic liquids (Bio-ILs), have gained attention for their high ionic conductivity and biocompatibility. These materials, composed of organic cations and anions, can provide tunable conductivity while maintaining structural stability. Choline-based ionic liquids have been incorporated into bioelectronic devices to facilitate charge transport in cell culture systems [76, 77]. Additionally, ionic liquids are being explored as conductive dopants in polymer matrices to enhance flexibility and electrochemical stability.

## Integration of conductive materials in sustainable biomedical research

A major challenge in integrating conductive materials in biomedical research is ensuring biodegradability and sustainability. Many synthetic conductive polymers and carbon-based materials are non-degradable, raising concerns about their long-term environmental impact. Recent advancements focus on eco-friendly alternatives, such as lignin-derived carbon nanofibers, biodegradable conductive polymers, and green-synthesized metallic nanoparticles [78]. Future research aims to balance electrical functionality with environmental responsibility, paving the way for next-generation sustainable conductive biomaterials in in vitro modeling. By expanding the scope of conductive materials, in vitro models can better mimic bioelectric environments, improving the physiological relevance of tissue engineering, drug screening, and disease modeling applications. Table 1 compares the different reported conductive materials in the in vitro models. This interdisciplinary approach bridges the gap between bioelectronics, materials science, and regenerative medicine, setting the foundation for more sustainable and effective biomedical research.

**Table 1:** Comparison of conductive biological materials in in vitro models

Material	Conductivity (S/cm)	Mechanism of conductivity	Mechanical Properties	Degradation Rate	Application	Reference
Polyaniline (PANI)	10⁻^4^–10⁻^1^	Conductivity via electron movement along conjugated polymer chains, enhanced by doping with protons or cations	Moderate stiffness; brittle in dry state	Weeks to months	Neural and cardiac tissue scaffolds	[79]
Polypyrrole (PPy)	10⁻^3^–10⁻^1^	Conductivity from delocalized π-electrons along the polymer backbone, with charge carriers introduced by doping	Flexible; tunable mechanical strength	Weeks to months	Nerve regeneration, cardiac scaffolds	[80]
PEDOT:PSS	10⁻^3^–10⁰	Conductivity due to the conjugated π-system in PEDOT, PSS improves solubility but reduces biofunctionality; conductivity modulated by modification or removal of PSS	Soft, hydrophilic, and elastic	Poorly biodegradable, modification required	Neural tissue scaffolds, bioelectronics	[39]
Graphene-based composites	10⁰–10^2^	Conductivity from delocalized π-electrons in graphene's conjugated structure; forms conductive networks that enable efficient charge transfer	High mechanical strength, flexible	Slow degradation, long-term stability	Electrodes, biosensors, conductive scaffolds	[81]
Carbon Nanotubes (CNTs)	10^2^–10^3^	High conductivity due to delocalized π-electrons along the carbon–carbon bonds in CNTs facilitates charge transfer via efficient electron movement along the tube surface	High tensile strength, stiff	Poor biodegradability	Neural and muscular tissue engineering	[82]
Lignin-derived Carbon Fibers	10⁻^3^–10⁰	Conductivity from graphitic structures formed during lignin pyrolysis; electron movement through conjugated carbon–carbon bonds	Sustainable, moderate mechanical properties	Slow degradation	Sustainable conductive scaffolds	[82, 83]
Chitosan-PPy composites	10⁻^5^–10⁻^2^	PPy provides conductivity through its conjugated structure; chitosan enhances biodegradability and biocompatibility while contributing to the composite's overall properties	Soft, biodegradable	Days to weeks	Nerve tissue scaffolds, wound healing	[84, 85]
Gelatin-MWCNT composites	10⁻^2^–10⁰	CNTs provide conductivity through electron delocalization along their carbon bonds; the gelatin matrix improves mechanical properties and biocompatibility	Biocompatible, tunable mechanical properties	Moderate degradation	Conductive scaffolds for cardiac tissue models	[86, 87]
Silk fibroin-PPy scaffolds	10⁻^3^–10⁻^2^	PPy contributes to conductivity through delocalized π-electrons in its conjugated backbone; silk fibroin adds mechanical strength and biodegradability	Elastic, tunable porosity	Biodegradable	Nerve regeneration, cardiac scaffolds	[88, 89]
Hyaluronic acid-PEDOT hydrogels	10⁻^3^–10⁰	PEDOT provides conductivity due to its conjugated π-system; HA enhances hydrophilicity and elasticity, aiding in biocompatibility and conductivity	Hydrated, elastic	Slow degradation	Brain-on-a-chip, neuronal interfaces	[73, 90]
Alginate-GO (Graphene Oxide) hydrogels	10⁻^4^–10⁻^2^	Graphene oxide contributes conductivity through its conjugated structure and formation of conductive networks; alginate provides mechanical support and biocompatibility	Highly porous, mechanically strong	Moderate degradation	Tissue engineering, drug delivery	[91, 92]

## Conductive materials in 3D in vitro models and their influence on cell behavior

The integration of conductive materials in 3D in vitro models has enabled the development of biomimetic platforms that better replicate the electrophysiological properties of native tissues. The impact on cellular behavior and signaling pathways by means of electrical stimulation (ES) applied through conductive scaffolds significantly influences cellular processes such as proliferation, differentiation, and migration. These effects are mediated by key signaling pathways, which include the following:

1. PI3K/Akt pathway: Electrical stimulation has been shown to activate the phosphoinositide 3-kinase (PI3 K)/Akt pathway, which plays a critical role in promoting cell survival, proliferation, and differentiation. Studies have demonstrated that conductive substrates enhance the phosphorylation of Akt, leading to improved cell viability and tissue regeneration, particularly in cardiac and neural applications [93, 94].
2. MAPK/ERK pathway: The mitogen-activated protein kinase/extracellular signal-regulated kinase (MAPK/ERK) pathway is another critical mechanism influenced by electrical stimulation. This pathway is associated with enhanced cell proliferation and differentiation, particularly in neuronal and muscle tissue engineering [95]. Conductive hydrogels embedded with graphene or PEDOT:PSS have been reported to promote ERK phosphorylation, leading to improved neurite outgrowth and muscle differentiation [96].
3. Wnt/β-catenin pathway: Electrical stimulation through conductive scaffolds can also regulate the Wnt/β-catenin signaling pathway, which is crucial for stem cell differentiation and tissue development [97]. This pathway has been particularly relevant in studies involving conductive polypyrrole (PPy) hydrogels, where electrical stimulation facilitated stem cell differentiation into neuronal and osteogenic lineages [98, 99].
4. Voltage-gated ion channels and calcium signaling: Conductive materials facilitate cell communication through voltage-gated ion channels. The depolarization induced by electrical stimulation activates calcium channels, increasing intracellular Ca^2^⁺ levels, which in turn influences cellular responses such as differentiation and synaptic plasticity [100]. This effect has been observed in conductive scaffolds for cardiac tissue engineering, where the controlled electrical stimulation enhanced cardiomyocyte alignment and functional maturation [101].

A notable study by Yan et al. [102] developed a conductive polypyrrole-chitosan hydrogel designed for cardiac tissue engineering. The hydrogel provided a physiologically relevant conductive microenvironment that supported the functional maturation of human cardiomyocytes. Electrical stimulation through the hydrogel led to upregulated expression of cardiac-specific genes (e.g., GATA4, Nkx2.5) and enhanced intercellular connectivity via connexin- 43 (Cx43) upregulation, facilitating synchronized contraction in 3D cultures. This study highlights how conductive hydrogels improve cellular organization and activate key molecular pathways involved in cardiac tissue formation.

## Integration of sustainability and conductivity

Though as beneficial to the conductive properties of biomaterials that these conductive additives are, another potential issue presents itself in the sustainability of these materials. As mentioned above, carbon-based materials are often used as conductive additives. Traditionally, the use of carbon fibers centered primarily on the aerospace, military, automobile, or energy industries, but their use in biomedical applications is increasing, particularly in the conductive biomaterials research field. Taking the example of carbon fibers (CF), their production poses many environmental and sustainability implications, primarily focused on the very high temperatures required to produce the CFs, which is associated with high carbon footprints. The raw material precursor, which traditionally is polyacrylonitrile (PAN), is also a synthetic polymer. Novel ways are emerging to combat such sustainability issues, primarily in the use of sustainable biobased materials as precursors in the production of CF instead of PAN, with lignin and cellulose chosen at the forefront of this novel research. Both lignin and cellulose are the most abundant components of lignocellulosic biomass, present in the cell wall of pith, roots, fruit, buds and bark. Lignin, in particular, is a non-valorized waste product of the paper industry, possessing an amorphous structure [103]. The Collins’ group at the University of Limerick, amongst others, have utilized lignin and its properties in the production of more sustainable carbon fibers [104], which can be further integrated as a sustainable conductive biomaterial in the conductive in vitro models.

Cellulose can also be utilized for similar purposes to improve the sustainability of the materials used in the in vitro models. In a study by Kuzmenko et al. [64], cellulose was electrospun and carbonized to produce CNFs mats with the results compared to the application of multi-walled CNT (MWCNTs) on the electrospun cellulose mats on the biological responses of in vitro neuronal networks. Overall, the results showed more positive results for the carbonized cellulose rather than the addition of the MWCNTs, ranging from improved mechanical properties to conductivity increases resulting from the lack of pure uncovered CNT cellulose fibers which impede the conductivity in the carbonized cellulose group, to increased hydrophobicity of the material caused by the small amounts of polar groups on the surface. Cytotoxicity testing with L929 fibroblasts also revealed positive biocompatibility results for the carbonized cellulose, while the cellulose/MWCNTs showed potential cytotoxic effects on the fibroblast cells. Similar results were observed when testing SH-SY5Y cells, with the best cellular performance and extension of neuritis observed on the carbonized scaffolds [105].

### Green synthesis approaches for conductive biomaterials

Green chemistry strategies have also been increasingly applied to fabricate sustainable conductive biomaterials, focusing on the following:

- *Bio-based graphene production*: Graphene, a widely used highly conductive material, is traditionally produced using chemical vapor deposition (CVD) or Hummers’ method, both of which involve hazardous reagents. Newer techniques use plant-derived precursors and enzymatic methods to synthesize biocompatible and environmentally friendly graphene [106, 107].
- *Biotemplating for conductive scaffolds:* Natural templates such as algae, fungi, and bacterial biofilms are now used to fabricate hierarchically structured, conductive biomaterials with tunable properties, reducing the reliance on synthetic polymers [60].

### Environmental and biomedical impact of sustainable conductive materials

- *Life cycle analysis (LCA) and carbon footprint reduction:* LCA studies indicate that replacing petroleum-based conductive materials with bio-derived carbon nanomaterials can lower greenhouse gas emissions and improve material biodegradability [61].
- *Cytocompatibility and reduced toxicity*: Studies show that green-synthesized conductive materials, such as cellulose-graphene hybrids and silk-PEDOT composites, significantly reduce inflammatory responses and cytotoxicity compared to traditional conductive polymers [108].
- *Circular economy integration*: The development of biodegradable and recyclable conductive biomaterials aligns with circular economy principles, ensuring sustainable use in biomedical applications while minimizing environmental impact [109].

###  Towards fully sustainable conductive biomaterials

The future of conductive biomaterials lies in developing fully renewable, biodegradable, and self-healing electronic biomaterials that can be naturally degraded or recycled after use. Emerging trends include the following:

- *Bioelectronics and implantable devices*: Conductive biomaterials with self-healing, bioresorbable, and flexible properties will be essential for next-generation neural interfaces, biosensors, and bioactuators [110].
- *Smart 3D bioprinting of conductive scaffolds*: The integration of bioinks derived from conductive biopolymers will enable the fabrication of patient-specific, sustainable tissue models with precise electrical properties [111, 112].
- *Hybrid organic–inorganic conductive materials:* Combining naturally derived conductive polymers with biodegradable metal–organic frameworks (MOFs) could lead to highly tunable, eco-friendly conductive platforms [113].

Researchers can develop high-performance, eco-friendly conductive biomaterials that align with FAIR data principles, sustainability goals, and biomedical safety standards by integrating renewable resources, green synthesis techniques, and biodegradable materials. These innovations ensure that next-generation conductive in vitro models are environmentally responsible while maintaining superior functional properties for biomedical research and applications (Table 2).

**Table 2:** Summary of relevant in vitro models for conductive biological materials

Sn	Conductive materials	Conductivity mechanism	Cell type used	Application	Advantages	Reference
1	Poly-l-Lysine-PANI nanotubes membranes	Charge transfer, Electron mobility	Rat CMs	culture of cardiac cells	electroactivity, biocompatibility, cardiac regeneration	[114]
2	PLCL, PANI electrospun membranes	Electron hopping, Charge transfer	Human fibroblasts, NIH- 3 T3, C2 C12	Tissue engineering platform"	Enhanced conductivity, cell adhesion	[115]
3	Carbon nanotubes (CNT) and poly(3,4-ethylenedioxythiophene) (PEDOT)	Electropolymerization	Neuroblastoma-derived SH-SY5Y cells	Tissue regeneration for neurodegenerative disorders; 3D scaffolds for neuronal differentiation	High conductivity and biocompatibility promote neuronal differentiation and enhance tissue regeneration in 3D	[116]
4	Silk fibroin (SF) and graphene-based nanomaterials (GBNs)	Interfacial interactions between SF and GBNs	stem cells (hPDLSCs)	Tissue engineering, wearable devices	Enhanced mechanical and electrical properties, biocompatibility, and biomedical potential	[117]
5	PGLD, PANI nanotubes membranes	Electroactive properties and microcurrent	Cho cells, neonatal rat CMs	Cardiac tissue engineering	Good biocompatibility	[118]
6	PU-AP/PCL porous scaffold	Aniline pentamer-based conductivity	Neonatal rat CMs	Cardiac tissue regeneration	Biodegradable, electroactive, enhances cardiomyocyte growth	[119]
7	PANI/PCL patch	Percolative network of polyaniline fibers	hMSCs	Cardiac tissue regeneration	Differentiation of hMSCs to CM-like cells	[120]
8	PCL, PPy films		HL- 1 murine CMs	Cardiac tissue engineering	Enhanced cell adhesion, functional properties, faster calcium wave propagation	[121]
9	Chitosan, PPy porous membranes	Polypyrrole grafted onto chitosan membrane	NRVMs, rat MI model	Myocardial repair, cardiac patch	Enhanced cell viability, angiogenesis, improved cardiac function	[122]
10	PCL, gelatin, PPy electrospun membranes	Polypyrrole incorporation in PCL/gelatin	Rabbit primary CMs	Cardiac tissue regeneration	Balanced conductivity, mechanical properties, enhanced cell proliferation	[123]
11	PEG/PEDOT:PSS hydrogels	In situ polymerization of PEDOT within PEG-PSS hydrogel	H9c2	High conductivity, biocompatibility, supports cell adhesion and proliferation	In situ polymerization of PEDOT within PEG-PSS hydrogel	[124]
12	GelMA/PEDOT:PSS hydrogels	PEDOT:PSS complex within GelMA	C2 C12	Electroconductive biomaterial for tissue stimulation	Tunable properties, biocompatibility, supports cell viability	[125]
13	Collagen/alginate/ PEDOT:PSS hydrogels	PEDOT:PSS in biohybrid hydrogel	CMs, hiPSCs-CMs	Cardiac tissue engineering	Good cell viability, proliferation, and Improved electrical coupling, enhanced cardiomyocyte maturation, prevents arrhythmia	[126]
14	PCL/PEDOT:PSS microfibrous scaffold	Conductive fibers via EHD printing	H9c2, primary CMs	Cardiac tissue regeneration	Enhanced alignment, conductivity, and cellular proliferation	[127]
15	Chitosan, PANI auxetic patch	Polyaniline-chitosan composite	murine neonatal cardiomyocytes in vitro and rat hearts for ex vivo	Myocardial infarction treatment	Auxetic design, cytocompatible, conforms to heart movements	[128]
16	PPy, HPAE, gelatin hydrogel	Fe3 + -triggered polymerization of pyrrole and dopamine Used Cell Lines: Not specified	Mouse fibroblast (L929) cells and bone marrow stromal cells (BMSCs)	Myocardial infarction treatment	Suture-free, conductive, enhances cardiac function and revascularization	[129]
17	poly- 3-amino- 4-methoxybenzoic acid (PAMB)-G hydrogel	Self-doped conductive polymer (PAMB) in gelatin	Cardiomyocytes (CMs)	Cardiac tissue repair	Enhanced electrical conduction, synchronized CM contraction, reduced arrhythmia	[130]
18	Cellulose, multi-walled carbon nanotubes	Carbonization and multi-walled carbon nanotubes	SH-SY5Y neuroblastoma cells	Neural tissue engineering, drug screening	Enhanced cell attachment, growth, differentiation, neural network formation	[105]
19	Gelatine and carbon nanofibers (CNFs)	CNF incorporation enhances conductivity	NIH/3 T3 fibroblasts	Cardiac, neuronal tissue engineering	Improved mechanical performance, cellular proliferation, and shear-thinning behavior for 3D printing	[131]
20	Germanium phosphorus (GeP3) nanosheets	Electromagnetic induction effect for wireless electrical stimulation	Neural stem cells (NSCs)	Spinal cord injury repair, tissue engineering	Biodegradable, enhances neural differentiation, improves locomotor function recovery	[132]
21	Carbon nanotubes (CNTs)	CNTs provide electroconductivity, reduce resistivity	PC12	Spinal cord injury repair	Improved cell viability, guided neuronal behavior, enhanced mechanical stability	[133]
22	Pyrrole (PPy) and hyaluronic acid (HA)	Covalent bonding between HA and pyrrole, polymerization of PPy	Fibroblasts	Biomaterials, drug delivery, stem cell culture	Biocompatibility, biodegradability, enhanced structural stability	[90]
23	Lignin and cellulose	Electrical conductivity properties of Ag-Lignin NPs	mouse fibroblast (L929)	Wound healing, antimicrobial treatment	Self-healing, antimicrobial, promotes wound healing, enhances tissue regeneration	[134]
24	Reduced graphene oxide (rGO)	Electrical conductivity through aligned fibers	Human induced pluripotent stem cell-derived cardiomyocytes (hiPSC-CMs)	Cardiac repair, drug screening	Enhanced electrical conductivity, oxidation resistance, improved cell maturation	[135]
25	Glassy carbon electrodes (GCE)/nitrogen doped porous rGO	Differential pulse voltammetry (DPV)	Human serum	Cardiac biomarker detection	High sensitivity, label-free, selective detection	[136]
26	Indium–Tin Oxides electrode/Manganese doped CdS sensitized graphene/Cu_2_MoS_4_	Charge transfer improvement	Human serum	Cardiac biomarker detection	High sensitivity, selectivity, stability	[137]
27	rGO/Gelatin methacryloyl (GelMA) hybrid hydrogel	Electron transfer through graphene networks	Cardiomyocytes	Cardiac tissue engineering	Improved cell viability, proliferation, maturation, contractility	[138]
28	rGO–reinforced gellan gum hydrogel	Electron transfer through graphene networks	Rat myoblasts (H9 C2)	Myocardial infarction repair	Improved mechanical properties, injectability, biocompatibility	[139]
29	GO/silk fibroin hydrogel	Electron transport through graphene sheets	Endothelial progenitor cells	Myocardial infarction regeneration	Biocompatibility, self-healing, enhanced cell delivery	[140]
30	Hydrazide functionalized/MWCNT/pericardial matrix hydrogel	Electron transport via carbon nanotubes (MWCNTs) and pericardial matrix interactions	hiPSC-derived cardiomyocytes	Cardiac tissue engineering	Improved cell maturation, alignment, contraction	[141]
31	Polyacrylonitrile (PAN), polyaniline (PANI), carbon nanotubes (CNTs)	Enhanced electroconductivity via CNT incorporation	mesenchymal stem cells (MSCs), neural stem cells (NSCs), or induced pluripotent stem cells (iPSCs)	Neural repair, stem cell differentiation	Biomimetic scaffolds, guided neural differentiation	[142]
32	Collagen, polypyrrole (PPy)	Electroconductive properties from PPy incorporation	Neural stem cells (NSCs)	Spinal cord injury (SCI) repair, neural regeneration	Biocompatible, enhanced neural differentiation, electrical stimulation	[143]
33	Polyethylene glycol (PEG), silver nanowires (AgNW)	Conductive AgNW network in PEG hydrogel	Neuronal stem cells (NSCs)	Neurodegenerative disease treatment, NSC therapy	Neurite guidance, flexible implant	[144]
34	Carbon nanomaterials, alginate hydrogel	Carbon nanomaterials enhance conductivity, support electrical stimulation	Neural progenitor cells (NPCs), astrocytes, oligodendrocytes	Neural interfaces, biohybrid electrodes	3D neural networks, tunable properties, long-term support	[145]
35	Polypyrrole (PPy), gelatin, oxidized xanthan gum (OXG)	electron delocalization reversible redox reactions for charge transfer	Cardiomyocytes (rat model)	Myocardial infarction treatment, tissue regeneration	Self-healing, conductive, and biocompatible and improves cardiac function	[146]

## Future perspectives

Growing awareness of the need for sustainable research shapes the future of tissue engineering and regenerative medicine (TERM) [147, 148]. Researchers are increasingly focused on developing eco-friendly and sustainable (bio)materials aligned with green chemistry principles, aiming to minimize environmental impact without sacrificing functionality [149–151].

A promising area within TERM is the development of advanced in vitro models, including 3D tissue constructs and organ-on-a-chip platforms, which can offer more physiologically relevant systems for studying human biology and disease [152, 153]. Hydrogel-based microfluidic technologies and bioprinting technologies have the potential to transform in vitro 3D cell/tissue culture and modeling, offering a closer match to the mechanical properties of native cardiac tissue than PDMS-based models [154, 155]. However, the traditional hydrogel-based microfluidic platforms present device deformation, poor structural stability/reproducibility due to swelling, and a limited range of rigidity, which threatens their future applicability. However, Oliveira et al. have proposed a pioneering and biomimetic soft to hard microfluidic platform for 3D cell culture with superior mechanical performance which makes advantage of the unique properties of the enzymatically-crosslinked fibroin hydrogels [156].

A notable innovation also comprises the integration of structurally stable conductive biomaterials to simulate electrical activity, which is critical for replicating the microenvironment and functions of tissues like the heart, brain, and nervous system [102, 157–159]. The aforementioned advances in these conductive biomaterials have enabled more realistic models that closely emulate physiological reactions, enhancing studies of disease mechanisms, drug responses, and drug discovery [160]. This advancement brings in vitro 3D models closer to mimicking native tissue behavior, substantially increasing their accuracy and utility as preclinical testing tools [161].

In bioprinting, a shift toward renewable and recyclable biomaterials/bioinks has emerged, aiming to improve sustainability and reduce the environmental impact of medical research [162]. Currently, conductive materials have become increasingly important within this sustainable framework, particularly for their applications in biosensors and other biomedical devices [163, 164].

Conductive bioinks are a specialized type of ink used in bioprinting that incorporates electrically conductive materials to enable the printed structures to conduct electricity. By enabling electrical conductivity within printed structures, they facilitate the development of functional cardiac and neural tissues, bioelectronic interfaces, and advanced drug testing platforms. Their ability to integrate electrical stimulation and sensing capabilities offers exciting opportunities for creating physiologically relevant models and innovative devices for regenerative medicine and personalized healthcare [43, 111, 165].

Moreover, sustainable conductive polymers like polyaniline, polypyrrole, and conductive hydrogels with graphene derivatives used alone or in combination with other biocompatible polymers present new possibilities for creating biosensors embedded within bioprinted tissues [166, 167]. By integrating these materials, researchers can develop 3D bioprinted organs-on-a-chip with embedded sensing capabilities for real-time monitoring of cellular activities, drug screening, and microenvironmental conditions [168]. This advancement could greatly enhance the functionality of in vitro models, fostering innovative diagnostic and therapeutic applications in regenerative medicine [152, 168]. In the particular area of cardiology, new diagnostic solutions as alternatives to traditional electrocardiogram, cardiac imaging, and genetic testing can be envisioned.

More advanced biofabricated 3D in vitro models will increasingly integrate patient-derived cells with biodegradable scaffolds or microfluidic devices composed of environmentally friendly materials, in the future [22, 169]. Conductive biomaterials will further enable high-throughput screening and patient-specific monitoring of physiological responses in real-time [170]. By incorporating microfluidics and bioelectronic sensors an unprecedented accuracy in predicting drug efficacy and safety profiles will be achieved, thus helping to advance personalized medicine [171].

The societal implications of these advancements can be profound. By means of offering more accurate, ethical, and sustainable alternatives to traditional 2D models and animal testing, the biomedical field can advance scientific knowledge and address global challenges in sustainability and healthcare [158, 159, 172, 173]. This convergence of innovation promises a future where biomedical technologies are more effective and environmentally responsible.

Developing and applying conductive materials in biomedical settings require rigorous regulatory evaluation to ensure biocompatibility and safety. One significant regulatory challenge is the long-term stability and degradation of conductive polymers such as polypyrrole (PPy) and polyaniline (PANI) in physiological environments. These materials must meet regulatory agency standards, such as the U.S. Food and Drug Administration (FDA) and the European Medicines Agency (EMA), particularly concerning their biodegradability, cytotoxicity, and immunogenicity [174].

For example, PEDOT:PSS, has faced concerns due to the poor biofunctionality of the polystyrene sulfonate (PSS) component [175]. Recent studies have demonstrated that modifying PEDOT to remove PSS, replacing it with biocompatible stabilizers like poly(diallyldimethylammonium chloride) (PDADMAC) and hyaluronic acid (HA), can significantly enhance its biocompatibility while maintaining conductivity [39, 176]. Carbon-based conductive materials, such as graphene and carbon nanotubes (CNTs), also present concerns about biological systems'potential cytotoxicity, oxidative stress, and inflammatory responses. The ISO 10993 standards for the biological evaluation of medical devices provide guidelines for testing these materials for hemocompatibility, genotoxicity, and chronic toxicity before approval for clinical use. Future regulatory frameworks may increasingly emphasize life cycle analysis (LCA) to assess these materials’ sustainability and environmental impact, ensuring their application addresses biomedical functionality and ecological responsibility.

## Conclusion

Conductive biological materials and bioinks hold immense potential to transform in vitro 3D models by introducing dynamic bioelectronic and sensing capabilities. To realize this potential, research efforts and funding initiatives targeting projects involving the use of next-generation sustainable and conductive (bio)materials in in vitro heart, brain, and nervous system models should be prioritized, in coming years. If these efforts are realized, a new era of biomedical research will emerge with principles of sustainability and technological innovation for healthcare.

## Data Availability

My manuscript has no associated data.
